# Survival of patients with metastatic breast cancer: twenty-year data from two SEER registries

**DOI:** 10.1186/1471-2407-4-60

**Published:** 2004-09-02

**Authors:** Patricia Tai, Edward Yu, Vincent Vinh-Hung, Gábor Cserni, Georges Vlastos

**Affiliations:** 1 University of Saskatchewan, Faculty of Medicine, Saskatoon; Department of Radiation Oncology, Regina, Canada; 2 Radiation Oncology Program, London Regional Cancer Centre, University of Western Ontario, London, Ontario, Canada; 3 AZ-VUB, Oncologisch Centrum, Jette, Belgium; 4 Bács-Kiskun County Teaching Hospital, Surgical Pathology, Kecskemét, Hungary; 5 Geneva University Hospitals, Department of Gynecology and Obstetrics, gynecologic oncology and senology, Geneva, Switzerland

## Abstract

**Background:**

Many researchers are interested to know if there are any improvements in recent treatment results for metastatic breast cancer in the community, especially for 10- or 15-year survival.

**Methods:**

Between 1981 and 1985, 782 and 580 female patients with metastatic breast cancer were extracted respectively from the Connecticut and San Francisco-Oakland registries of the Surveillance, Epidemiology, and End Results (SEER) database. The lognormal statistical method to estimate survival was retrospectively validated since the 15-year cause-specific survival rates could be calculated using the standard life-table actuarial method. Estimated rates were compared to the actuarial data available in 2000. Between 1991 and 1995, further 752 and 632 female patients with metastatic breast cancer were extracted respectively from the Connecticut and San Francisco-Oakland registries. The data were analyzed to estimate the 15-year cause-specific survival rates before the year 2005.

**Results:**

The 5-year period (1981–1985) was chosen, and patients were followed as a cohort for an additional 3 years. The estimated 15-year cause-specific survival rates were 7.1% (95% confidence interval, CI, 1.8–12.4) and 9.1% (95% CI, 3.8–14.4) by the lognormal model for the two registries of Connecticut and San Francisco-Oakland respectively. Since the SEER database provides follow-up information to the end of the year 2000, actuarial calculation can be performed to confirm (validate) the estimation. The Kaplan-Meier calculation for the 15-year cause-specific survival rates were 8.3% (95% CI, 5.8–10.8) and 7.0% (95% CI, 4.3–9.7) respectively. Using the 1991–1995 5-year period cohort and followed for an additional 3 years, the 15-year cause-specific survival rates were estimated to be 9.1% (95% CI, 3.8–14.4) and 14.7% (95% CI, 9.8–19.6) for the two registries of Connecticut and San Francisco-Oakland respectively.

**Conclusions:**

For the period 1981–1985, the 15-year cause-specific survival for the Connecticut and the San Francisco-Oakland registries were comparable. For the period 1991–1995, there was not much change in survival for the Connecticut registry patients, but there was an improvement in survival for the San Francisco-Oakland registry patients.

## Background

Prospective trials have the disadvantages of requiring a long time to complete, and using highly selected patient subgroups in tertiary centers. While one waits for the results to mature, this delays additional research to improve treatment. If there were a method that allowed earlier prediction of the results of prospective trials, advances in cancer treatment could be attained within a shorter time period.

There is a parametric lognormal model, proposed by Boag [1-3] that had been retrospectively validated in the literature, and could be used prospectively for clinical trials to predict long-term survival rates several years earlier than would otherwise be possible using the standard life-table/actuarial Kaplan-Meier method of calculation [4].

The prognosis for metastatic breast cancer is generally poor and therefore it is believed that statistical prediction models for long-term survival rates are not needed. Nevertheless, specific subgroups of metastatic breast cancer patients exist, for which depending on the treatment given, the prognosis is improved so that some patients can survive for some time, particularly for those with limited organs involvement such as involvement with bone and/or skin only. In this situation, for which the present study was relevant, a prediction model, even for metastatic breast cancer, can be useful.

Breast cancer, among other cancers, has the highest incidence in women, and many studies are currently in progress to assess treatment regimens. If, even for a subgroup of patients, the 10- and 15-year survival rates can be predicted from follow-up data available only 3 years after a 5-year diagnosis period, this would be a useful means of obtaining study results earlier than would otherwise have been possible. For example, a 15-year survival rate calculated by the Kaplan-Meier method requires at least some patients to have been followed for 15 years. In addition prediction model such as the lognormal model can also be used to review the progress of treatment results for a specific period from a treatment center, and to compare that with another specific period of the same treatment center to evaluate the potential impact for any possible change in treatment policy or guideline.

Boag's lognormal model for long-term cancer survival rates has been available for use for some 50 years. When the lognormal model was first proposed in the 1940s, it was difficult to implement because of a lack of computing power, and lack of good quality long-term follow-up data from cancer registries. Since 1970s the model has been used by some authors in breast cancer, cervix uteri cancer, head and neck cancer, intraocular melanoma, choroidal-ciliary body melanoma, and small cell lung cancer [5-10]. Currently, although the computing power is sufficient, good quality follow-up data on a sufficient number of patients are seldom available, and it can be a limitation for its application. Large data registry such as the Surveillance, Epidemiology, and End Results (SEER) data [11] with good long-term follow-up data available can overcome this potential limitation.

## Methods

Between 1981 and 1985, 782 and 580 female patients of metastatic breast cancer were extracted respectively from the Connecticut and San Francisco-Oakland registries from the SEER database using SEER*Stat 5.0 software. The two registries were chosen because they are two of the earliest registries, with a large population. The data used were survival time, vital status, cause of death, age at diagnosis, and race.

The cause-specific survival was defined as the interval from the date of diagnosis to the date of death from breast cancer or the last follow-up date for censoring purposes, if the patient was alive and still being followed at the time of analysis. The survival time of the uncured group of patients who died of breast cancer had been verified to follow a lognormal distribution previously [12].

Next, between 1991 and 1995, 752 and 632 female patients of metastatic breast cancer were extracted respectively from the two registries. The data were used to estimate the 15-year cause-specific survival rates before the year 2005. To be comparable, for both the 1981–1985 and 1991–1995 cohorts, the staging system used was the SEER historical system (classified as localized, regional, or distant, based on combined pathologic and clinical data). The choice of 1981–1985 and 1991–1995 has the advantage that the two time periods are not too far apart otherwise there would be too much changes of medical practice. These time periods have a minimum of 5 years follow-up.

The overall survival rates (OSR) of the two time periods were calculated using the Kaplan-Meier method. The actual relative survival rates (RSR) were calculated using SEER*Stat 5.0 software. The modified version of period analysis [13] was applied using the Hakulinen method [14] to obtain more up-to-date absolute survival rates (ASR) and relative survival rates (RSR) for comparison purpose with a computer program run by Microsoft Excel software.

### Validation of the lognormal model

The validation of the lognormal model has two phases. Phase 1 tests the goodness of fit to a lognormal distribution of the survival time of those cancer patients who died with their disease present, termed an uncured group with a fraction of 1-C, where C is the cured proportion of patients. The lognormal distribution is similar to the normal distribution in that if the variable in the normal is time t, the variable in the lognormal is the logarithm of t. In other words, the investigators attempt to show that the logarithm of the survival time follows a normal distribution. Phase 2 attempts to show that the lognormal model, using short-term follow-up data, can predict long-term survival rates comparable to those calculated by the Kaplan-Meier life-table method with long-term available. This model can be used to estimate long-term cause-specific survival rates (CSSR) by a maximum likelihood method (e.g., 10-year and 15-year survival rates) from only short-term follow-up data. The maximum likelihood method is used to estimate the CSSR at time τ, and is calculated as [C+(1-C)·Q]·100%, where Q is the integral of the lognormal distribution between the limits time τ and infinity.

The lognormal statistical model had been validated in stages III and IV breast cancer in a previous publication that survival rates could be estimated several years earlier than is possible using the standard life-table actuarial method [12]. The survival time of unsuccessfully treated cases could be represented by a lognormal distribution, the long-term survival rates were predicted by Boag's method using a computer program run by Microsoft Excel. In this parametric lognormal model, the standard deviation S was fixed, and only the two remaining parameters, mean M and proportion cured C, were kept floating when using the maximum likelihood method. Multiple iterations converged to a stable solution for C.

A 5-year period of diagnosis could be selected and patients followed as a cohort for an additional 3 years. The current study was for metastatic breast cancer patients treated between 1981 and 1985, with follow-up to the end of year 2000, making the series ideal for validating purposes. For example, for cases diagnosed during the 5-year period, prediction of the 15-year survival rate was made using data at the follow-up cutoff date of December 31, 1988 (i.e., 3 years after 1985). The 15-year survival rate prediction was then validated by Kaplan-Meier life-table calculations using the follow-up data available in 2000.

For metastatic breast cancer patients treated between 1991 and 1995, and follow-up to the end of year 2000, prediction of the 15-year survival rate was made using data at the follow-up cutoff date of December 31, 1998 (i.e., 3 years after 1995) before the year 2005.

## Results

From the cohort of 1981–1985 inclusively, 782 patients from the Connecticut registry were followed to the end of 1988. The lognormal model predicted the 15-year CSSR to be 7.1% (95% CI, 1.8–12.4). The 15-year CSSR was 8.3% (95% CI, 5.8–10.8) validated by the Kaplan-Meier calculation using actuarial follow-up data up to the end of year 2000.

From the cohort of 1981–1985 inclusively, 580 patients from the San Francisco-Oakland registry were followed to the end of 1988. The lognormal model predicted the 15-year CSSR to be 9.2% (95% CI, 3.9–14.5). The 15-year CSSR was 7.0% (95% CI, 4.3–9.7) validated by the Kaplan-Meier calculation using actuarial follow-up data up to the end of year 2000.

Using the same method, the cohort of 1991–1995 inclusively, 752 patients from the Connecticut registry were followed to the end of 1998. The lognormal model predicted the 10-year CSSR to be 12.6% (95% CI, 7.3–17.9). The 10-year CSSR was 11.3% (95% CI, 7.8–14.8) validated by the Kaplan-Meier calculation using actuarial follow-up data up to the end of year 2000. The lognormal model predicted the 15-year CSSR to be 9.1% (95% CI, 3.8–14.4), which cannot be validated before 2005.

For the cohort of 1991–1995 inclusively, 632 patients from the San Francisco-Oakland registry were followed to the end of 1998. The lognormal model predicted the 10-year CSSR to be 17.0% (95% CI, 12.1–21.9). The 10-year CSSR was 15.9% (95% CI, 11.4–20.4) validated by the Kaplan-Meier calculation using actuarial follow-up data up to the end of year 2000. The lognormal model predicted the 15-year CSSR to be 14.7% (95% CI, 9.8–19.6), which cannot be validated before 2005.

For the period 1991–1995, there was not much change of only about 2% absolute percentage point in the predicted 15-year CSSR for the Connecticut registry, but there was an improvement of about 6% absolute percentage points for the San Francisco-Oakland registry when compared with the period 1981–1985 15-year CSSR, which was validated by the Kaplan-Meier calculation. (Table 1)

For comparison purpose, the actual OSR and RSR were compared with the ASR and RSR obtained by the period analysis. (Tables 2 and 3) It was found that there were more patient survival improvements shown in the actual OSR and RSR for the San Francisco-Oakland registry, but not much for the Connecticut registry. However the period analysis results did not show such improvements.

## Discussion

### Lognormal model

Rutqvist studied the fit of Boag's lognormal model to the survival times of 8170 breast cancer cases reported to the Swedish Cancer Registry during 1961–1963. The model fitted the 1961–1963 data well for the entire case material and for patients aged less than 70 years. In this registry, the lognormal model did not fit the data for patients aged greater than 70 years, who were more likely to be censored because of coincidental causes of death. Another disadvantage stated by the author was that large number of patients was required to obtain estimates with reasonably small standard errors for breast cancer.

With another series of the Norwegian Cancer Registry of 14,000 breast cancer cases, Rutqvist *et al*. [15] deduced that lognormal is the best model because other models did not fit the observed survival in all stages, ages, and time periods (two-parameter models, such as exponential or extrapolated actuarial, or three-parameter models, such as sum of two exponential, exponential with shoulder, Weibull). Both the exponential and extrapolated actuarial models assume that the conditional relative survival is lowest immediately after treatment. With the lognormal model, the survival curve has a low initial mortality that rapidly increases to a maximum, with a slow decrease in the mortality after the maximum has occurred.

### Requirements for using the lognormal model

The lognormal model can only predict cause-specific survival, because other coincidental causes of death are too unpredictable (e.g., the rate of stroke). Therefore, overall survival cannot be predicted. The maximum likelihood method is the most accurate method for fitting the lognormal model with the smallest mean squared error. However, there are some requirements for its use. The maximum likelihood method fails to converge to a stable solution using the initial estimates if there is extensive censoring within the data. This occurs if patients are lost to follow-up or die from coincidental non-cancerous causes. The frequency of failure to yield a successful fit for lognormality was greater when one-fourth of cases were designated as lost to follow-up. Gamel *et al*. established a stable linear algorithm for fitting the lognormal model to survival data. To achieve convergence, some authors have fixed one or two parameters of the lognormal model to pre-selected values to simplify the iterative procedure required for convergence [6].

### Some prognostic factors follow lognormal distribution

Prognostic factors in patients with distant metastases at the time of diagnosis were investigated by Rudan *et al*. [16], and Chapman *et al*. [17], primary tumor size was a significant prognostic factor. Engel *et al*. [18] found that the number of metastatic cases and the time to metastasis depended on the tumor diameter at diagnosis. Cell growth is essential for the development of tumors. Tumor size is therefore the most important factor in describing tumor biology. As the tumor size increases, the probability of node-positivity increases. Another study group also found this correlation up to 5 cm [19]. Tubiana and Koscielny [20] have found a highly significant correlation between tumor size and the probability of distant metastasis. The distribution of tumor sizes at metastatic spread was *lognormal *with a median diameter equal to 3.5 cm. The patients were subdivided into 3 groups according to the histological grade. In each subgroup there was a significant correlation between tumor size and the probability of distant spread. The distributions were lognormal and the median size was markedly larger for grade 1 tumors.

A number of quantitative postmortem observations regarding the size distribution of metastases have been published [21-23]. These studies revealed a skewed distribution with a high proportion of smaller metastases, and a significant tail extending to the larger metastases, consistent with a lognormal distribution. The more detailed measurements from human liver metastases provided by Yamanami *et al*. were found to approximate the lognormal distribution reasonably well.

A hypothesis was proposed by Kendal [24] that the time available for the growth of metastases is normally distributed, presumably as a consequence of the summation of multiple independently distributed time intervals from each of the steps and of the Central Limit Theorem. For exponentially growing metastases, the corresponding size distribution would be lognormal; Gompertzian growth would imply a modified (Gompertz-normal) distribution, where larger metastases would occur less frequently as a consequence of a decreased growth rate. These two size distributions were evaluated against 18 human autopsy cases where precise size measurements had been collected from over 3900 macroscopic hematogenous organ metastases. The lognormal distribution provided an approximate agreement. Its main deficiency was a tendency to over-represent metastases greater than 10 mm diameter. These observations supported the hypothesis of normally distributed growth times, and qualified the utility of the lognormal and Gompertz-normal distributions for the size distribution of metastases.

Why is the lognormal model applicable to so many organ sites [3,6-10,12,25-36] (Table 4)? Boag's explanation for the lognormal survival time distribution was that if the patient was not cured by treatment, the length of the remaining survival time would be dependent principally on the growth rate of the tumor remnants. Pearlman [37] estimated the growth rates of breast cancer that recurred in the scar, assuming that the recurrence started from a single cell. He found that the growth rates were approximately lognormally distributed. Likewise, von Fournier *et al*. [38] found that the growth rates of breast cancers followed by serial mammography were lognormally distributed.

### Variation of survival rates over time

In order to determine whether current programs for the management of metastatic breast cancer have led to improved patient survival, Debonis *et al*. [39] determined the median survival times for five-year intervals of 849 patients admitted to the City of Hope National Medical Center with metastatic breast cancer from 1955 to 1980. Survival times in each of the clinical subsets remained unchanged during the period of observation, regardless of the therapeutic modalities included in the treatment regimens. The study indicates that changes in palliative therapy for metastatic breast cancer during the 25 years of observation have not influenced overall survival. On the contrary, Dickman *et al*. [40] studied the survival of cancer patients in Finland during the years 1955–1994. The 5-year RSR for distant metastases breast cancer had increased from 10% for the period 1955–1964 to 22% for 1985–1994.

The tumor registry at Yale-New Haven Hospital, which began recording data in 1920, was utilized by Todd *et al*. [41] to examine the ultimate outcome of all breast cancer patients who were initially diagnosed at Yale with metastatic breast cancer. The median survival of these patients increased steadily from 21 months in 1920 to 41 months in the decade from 1970 to 1980. The percentage of women actually surviving 5 years increased from 5% in the 1920s to approximately 25% in the 1960s. Despite the use of combination drug programs in the 1970s, the percentage of these patients remaining alive at 5 years remained near 25%. Firm conclusions cannot be made from a retrospective study spanning 60 years, although the trends depicted the lack of continued improvement indicate that the current therapeutic approach to metastatic breast cancer in that period may not result in dramatic improvement in overall survival.

### Geographical variation of survival rates

Farrow *et al*. [42] documented substantial geographical variation in patterns of treatment of cancer and other diseases. Because cancer treatment is not uniform nationwide in the States, survival following the diagnosis of cancer might also be expected to vary geographically. Survival data from the nine population-based registries in the SEER Program were analyzed for cancers of the stomach, colon, rectum, lung, breast, uterus, ovary, prostate, and bladder. The patients included all non-Hispanic white patients diagnosed with cancer of one of the selected sites during 1983–1991. Regional variation in crude five-year survival rates across the nine SEER areas was most marked for cancers of the uterus and prostate. For uterine cancer, for example, five-year survival ranged from 73.2% in Connecticut to 84.0% in Hawaii. Less marked variation was observed for cancers of the colon, rectum, and breast. For cancers of the bladder, ovary, stomach, and lung, survival rates five years after diagnosis were relatively invariant across the SEER areas.

Maggard *et al*. [43] also found that variations in the breast cancer mortality rates exist between states. A nearly 50% increase is observed between the states with the highest and lowest mortality rates. Adjusted analyses demonstrated that stage at presentation is a more important predictor of mortality variation than treatment differences. Goodwin *et al*. [44] examined breast cancer incidence, survival, and mortality in the 66 health service areas covered by the SEER program for women aged 65 and older at diagnosis. They found that there was considerable geographic variation in survival from breast cancer among older women, and this contributed to variation in breast cancer mortality. The elevated mortality in the Northeast is apparent only in older women [45]. For women aged 65 years and older, breast cancer mortality is 26% higher in New England than in the South, while incidence is only 3% higher. Breast cancer mortality for older women by state correlates poorly with incidence (r = 0.28).

The above-mentioned results are consistent with that from the present study: the Connecticut registry has lower CSSR than the San Francisco-Oakland registry for the period 1991–1995. The Connecticut cohort has median age at diagnosis of 66 (range 25–103), while the San Francisco-Oakland cohort has lower median age of 63 (range 26–96). It could be argued that new treatments evolved in the recent decade have improved the survival of the breast cancer patients, and younger patients benefit more than the older patients. Apart from treatment offered, changes of survival rates over time or geographical areas can be due to co-morbidities or other characteristics such as race, age, and differences in staging procedures.

## Conclusions

For the period 1981–1985, the 15-year cause-specific survival for the Connecticut and the San Francisco-Oakland registries were comparable. For the period 1991–1995, there was not much change in survival for the Connecticut registry patients, but there was an improvement in survival for the San Francisco-Oakland registry patients.

## List of abbreviations

CSSR: Cause-specific survival. SEER: Surveillance, Epidemiology, and End Results. OSR: Overall survival rate. ASR: Absolute survival rate. RSR: Relative survival rate.

## Competing interests

None declared.

## Authors contributions

PT: Data analysis and writing of manuscript. EY, VVH, GC, GV: Critical appraisal of manuscript.

All authors read and approved the final manuscript.

## Pre-publication history

The pre-publication history for this paper can be accessed here:



## Figures and Tables

**Table 1:** 10- and 15-year cause-specific survival rates for Connecticut and San Francisco-Oakland registries for 1981–1985 and 1991–1995 cohorts calculated by lognormal model (LN) and Kaplan-Meier (KM) method with 95% confidence intervals in brackets

	Connecticut				San Francisco-Oakland			
Year	1981–1985		1991–1995		1981–1985		1991–1995	
	LN	KM	LN	KM	LN	KM	LN	KM
10	11.5 (6.2–16.8)	11.0 (8.5–13.5)	12.6 (7.3–17.9)	11.3 (7.8–14.8)	12.3 (7.0–17.6)	9.7(6.8–12.6)	17.0 (12.1–21.9)	15.9 (11.4–20.4)
15	7.1 (1.8–12.4)	8.3 (5.8–10.8)	9.1 (3.8–14.4)	N.A.	9.2 (3.9–14.5)	7.0 (4.3–9.7)	14.7 (9.8–19.6)	N.A.

N.A. = not available

**Table 2:** 10- and 15-year OSR^a^, RSR^b^, ASR^c^, RSR^d ^for Connecticut registry for 1981–1985 and 1991–1995 cohorts with 95% confidence intervals in brackets

Connecticut								
Year	1981–1985				1991–1995			
	OSR^a^	RSR^b^	ASR^c^	RSR^d^	OSR^a^	RSR^b^	ASR^c^	RSR^d^

10	6.4 (4.6–8.2)	9.1 (6.7–11.5)	13.0 (12.2–13.8)	16.7 (15.7–17.7)	7.2 (3.7–10.7)	9.7 (6.4–13.0)	15.6 (14.6–16.6)	20.7 (19.5–21.9)
15	3.2 (2.0–4.4)	5.7 (3.5–7.9)	4.4 (4.0–4.8)	6.4 (6.0–6.8)	N.A.	N.A.	5.8 (5.4–6.2)	8.9 (6.2–9.5)

OSR^a ^= overall survival rate calculate by Kaplan-Meier method

RSR^b ^= relative survival rate calculated using SEER*Stat 5.0

ASR^c ^= absolute survival rate calculated by period analysis

RSR^d ^= relative survival rate calculated by period analysis

N.A. = not available

**Table 3:** 10- and 15-year OSR^a^, RSR^b^, ASR^c^, RSR^d ^for San Francisco-Oakland registry for 1981–1985 and 1991–1995 cohorts with 95% confidence intervals in brackets

San Francisco-Oakland								
Year	1981–1985				1991–1995			
	OSR^a^	RSR^b^	ASR^c^	RSR^d^	OSR^a^	RSR^b^	ASR^c^	RSR^d^

10	4.6 (2.8–6.4)	6.6 (4.2–9.0)	13.3 (12.3–14.3)	16.9 (15.7–18.1)	8.8 (5.7–11.9)	11.4 (7.9–14.9)	14.7 (13.7–15.7)	18.4 (17.2–19.6)
15	2.4 (1.2–3.6)	4.1 (1.9–6.3)	4.6 (4.2–5.0)	6.6 (6.0–7.2)	N.A.	N.A.	5.3 (4.9–5.7)	7.4 (6.8–8.0)

OSR^a ^= overall survival rate calculate by Kaplan-Meier method

RSR^b ^= relative survival rate calculated using SEER*Stat 5.0

ASR^c ^= absolute survival rate calculated by period analysis

RSR^d ^= relative survival rate calculated by period analysis

N.A. = not available

**Table 4:** Cancer sites with survival times demonstrated to follow the lognormal distribution in the literature as at year 2004

Cancer sites	Author
Head and neck cancer	Berg [25], Mould[7]
Nasal sinus cancer	Berg [25]
Mouth and throat cancer	Boag [3]
Mouth	Berg [25]
Thyroid	Tai*[26]
Larynx, tongue	Mould & Tai*[27]
Non small cell lung cancer	Berg [25]
Small cell lung cancer	Tai*[10]
Intraocular melanoma	Gamel [8]
Cutaneous melanoma	Gamel [9]
Breast cancer	Boag [3], Berg [25], Rutqvist [5,15], Gamel [28,29], Haybittle [30], Royston [31], Tai*[12]
Bone sarcomas	Berg [25]
Cancer of uterine cerivx	Mould & Boag*[6], Berg [25]
Ovarian cancer	Berg [25], Tai*[32], Royston [31]
Hypernephroma	Berg [25]
Bladder cancer	Berg [25]
Prostate Cancer	Mould & Tai*[33]
Gastric cancer	Berg [25], Maetani [34]
Lymphoma	Berg [25]
Chronic leukemia	Tivey [35]
Brain tumors	Berg [25]
42 SEER cancer sites	Tai [36]

SEER, Surveillance, Epidemiology, and End Results;

*, phase 2 validation also performed, i.e. concordance between Kaplan-Meier method and lognormal model.
